# Mechanism of microcirculation disturbance in diabetic nephropathy: A review

**DOI:** 10.1097/MD.0000000000045637

**Published:** 2026-04-17

**Authors:** Weikang Tang, Huixia Liu, Xuan Li, Siyao Deng, Changyu Gao

**Affiliations:** a School of Medicine, Tarim University, Alaer, Xinjiang, China; b Department of Prescription Science, Heilongjiang University of Chinese Medicine, Harbin, Heilongjiang, China.

**Keywords:** diabetic nephropathy, microcirculation, pathogenesis, signaling pathways

## Abstract

Diabetic nephropathy, as one of the most serious microvascular complications of diabetes, has an extremely complex pathogenesis. Currently, there are still no effective treatment methods available in clinical practice that can effectively prevent its pathological progression. In recent years, it has been discovered that the microcirculation system facilitates the exchange of substances between blood and tissues, profoundly influencing the homeostasis of the internal environment within the body. Abnormalities in renal microcirculation are closely related to the occurrence and development of diabetic nephropathy. Given the significant importance of improving renal microcirculation disorders in delaying the progression of diabetic nephropathy, by exploring the pathological association between microcirculation disorders and diabetic nephropathy, this paper elaborates on the “key node” role of microvascular lesions in the course of diabetic nephropathy, and conducts an analysis of the signaling pathways. It explains the interactions among cellular signal transduction, growth transformation factors, and oxidative stress pathways in microvascular lesions, providing a more comprehensive theoretical basis and research direction for the treatment strategy of diabetic nephropathy targeting improving renal microcirculation disorders.

## 1. Introduction

Diabetic nephropathy (DN) is one of the common microvascular complications of diabetes mellitus, and its pathological features are the thickening of renal tubular basement membrane and glomerular membrane, the accumulation of extracellular matrix (ECM) and progressive mesangial hypertrophy caused by microangiopathy.^[1]^ About 30% to 40% of diabetic patients will develop diabetic nephropathy. Increased urinary albumin excretion and decreased glomerular filtration rate (GFR) are used as diagnostic criteria to determine the development stage of the disease.^[2]^ Early morphological abnormalities of DN include early glomerular hypertrophy, mesangial matrix expansion and renal tubular injury. Further changes in renal tissue, including glomerulosclerosis and renal tubulointerstitial fibrosis, indicate that DN has progressed to end-stage renal disease (ESRD). Therefore, it is necessary to intervene in early DN patients.^[3,4]^ With the deepening of DN research, it is found that the dysfunction of microcirculation has a significant impact on the occurrence and development of diabetic microangiopathy. Studies have shown that,^[5]^ The reduction of renal microcirculation blood flow can lead to tissue hypoxia and damage. According to the characteristics of microcirculation disorders, improving microvascular circulation to protect the diabetic kidney has become the key to clinical targeted therapy and improving microcirculation of DN.

The essence of microcirculation disorders caused by DN is the obstruction of local blood microcirculation perfusion in the kidney, which changes the number and function of renal microvessels, and then leads to a series of changes in histopathological lesions and functional failure.^[6]^ Based on the study of microcirculation disorders in the development of DN, this article highlights the potential mechanisms and molecular targets, screens out the related signal transduction pathways, and elaborates the pathological effects of traditional Chinese medicine on renal microvascular circulation disorders, and provides a theoretical basis for the treatment of diabetic nephropathy.

## 2. Materials and methods

### 2.1. Search for relevant literature

PUBMED, PubMed, Web of Science and Google Scholar were used to search the literature on diabetic nephropathy and renal microcirculation in the past 15 years. The relevant articles on the pathogenesis of diabetes and the effect of microcirculation on renal injury were screened, read and summarized. Finally, the writing of the review was completed.

### 2.2. Ethical approval

The review did not involve human or animal studies and therefore did not require ethical approval.

## 3. Mechanisms of action of microcirculatory disturbances

**Figure 1.**
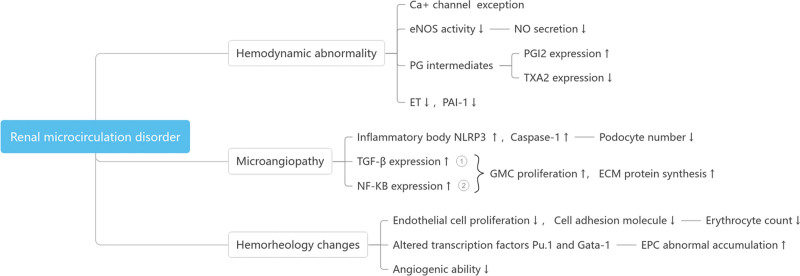
Mechanisms of action of renal microcirculation disturbances. ECM = extracellular matrix, EPC = erythroid progenitor cell, eNOS = endothelial nitric oxide synthase, GMC = glomerular mesangial cells, JAK/STAT = janus kinase-signal transducer and activator of transcription, NF-кB = nuclear factor-кB, NLRP3 = NOD-like receptor protein 3, NO = nitric oxide, PAI-1 = plasminogen activator inhibitor-1, PG = prostaglandins, PGI_2_ = prostacyclin, TGF-β=transforming growth factor-β, TXA_2_ = thromboxane A_2_.

Microcirculation refers to the blood circulation between tiny blood vessels, which is an important part of the systemic circulatory system and is mainly responsible for the material exchange between blood and tissues.^[7]^ Microcirculation changes are an objective reflection of renal related diseases and are characteristic. Studies have shown that,^[8]^ Microangiopathy occurs in DN patients before the impairment of renal function, suggesting that the dysfunction of microcirculation may occur throughout the development of DN, including hemodynamic abnormalities, microangiopathy and hemorheological changes. The final key culprit of renal failure is renal microcirculation dysfunction. The specific mechanism of action is shown in Figure 1.

### 3.1. Hemodynamic abnormalities

Hemodynamic abnormalities are the early manifestation of diabetic microcirculation disorders, which are closely related to the occurrence and development of DN. It is mainly characterized by the relaxation of the afferent arterioles and the constriction of the efferent arterioles, resulting in the increase of intrabellar pressure and microvascular blood flow. Studies have shown that early intervention with vasodilators in the course of the disease can not only correct the hemodynamic abnormalities in the renal microcirculation, but also help reverse renal function damage and restore normal renal perfusion, laying the foundation for delaying the progression of the disease.^[9]^ The clinical diagnosis of DN is usually based on glomerular hyperfiltration, hypertrophy and intermittent increased urinary protein excretion rate. When GFR is higher than 2 standard deviations of the normal population mean, it is considered to be hyperfiltration (HF).^[10]^ According to studies, it is found that with the appearance of GFR, glomerular capillary wall permeability and intraglomerular pressure increase, which in turn triggers microalbuminuria. In addition to albuminuria, abnormal GFR is a key marker for predicting the risk of ESRD and renal death in diabetic patients.^[11]^ By reducing glomerular pressure and reducing damage to the filtration barrier, drugs improve proteinuria excretion in DN patients, restore normal glomerular filtration rate, and reduce renal failure and disease progression.^[12-14]^ Podocytes and mesangial cells, which constitute the glomerular filtration barrier, express many different types of ion channels that regulate cellular function and response to the local environment.^[15]^

Renal microvessels mediate a variety of vasodilatory and vasoconstrictive receptors that can affect glomerular blood flow and capillary pressure. Persistent hyperperfusion of renal capillaries and increased glomerular pressure are the direct causes of glomerular hyperfiltration. In the hyperglycemic state, plasma osmolality increases, leading to an increase in renal blood volume, while persistent glomerular capillary hyperperfusion and glomerular hyperpressure accelerate the development of diabetic nephropathy.^[16]^ Renal microvessels mediate a variety of vasodilatory and vasoconstrictive receptors that can influence glomerular blood flow and capillary pressure. Among them, Ca^+^ channels can affect glomerular capillary tone. It can regulate the channel of cells, reduce the resistance of blood flow in the kidney, reduce the occurrence of GFR, and play a role in protecting the kidney.^[17,18]^ Nitric oxide (NO)^[19]^ and prostaglandins (PG)^[20]^ are vasoactive substances. NO is produced by L-arginine and oxygen molecules catalyzed by endothelial nitric oxide synthase (eNOS). Vascular endothelial cells regulate vascular tone by secreting endothelium-derived relaxation factor (NO), which plays a role in vasodilation by acting on renal glomerular arterioles^[21]^ eNOS phosphorylation plays an important role in regulating eNOS activity and NO production. Excessive NO secretion can change the permeability of endothelial cells, resulting in cell dysfunction and vascular injury. Regulation of vascular endothelial dysfunction; Prostacyclin (PGI_2_), a metabolite of arachidonic acid produced by vascular endothelial cells, not only dilates small blood vessels, but also inhibits platelet aggregation.^[22]^ Thromboxane A_2_ (TXA_2_), on the contrary, is produced by PG intermediate metabolite PGH2 and thromboxane synthase. It has strong vasoconstrictive and platelet aggregation effects. Recent studies have found that TXA_2_ may cause kidney injury.^[23]^ Arachidonic acid is metabolized to a variety of bioactive mediators such as PG and TXA through the COX pathway, which can improve hemodynamic abnormalities of renal microcirculation and increase renal blood flow.^[24,25]^ The RAAS plays an indispensable role in the homeostatic control of blood flow pressure, tissue perfusion and extracellular volume in the pre-DN stage, exerting effects on both systemic and local renal hemodynamics. Among them, angiotensin increases the level of transforming growth factor-β (TGF-β) mRNA in the mesangium, promotes the synthesis of matrix proteins (such as fibronectin, collagen, and laminin), and inhibits matrix degradation, leading to the accumulation of ECM, and also has a damaging effect on podocytes.^[26,27]^ In addition, plasminogen activator inhibitor-1, a major inhibitor of plasminogen activator, inhibits TGF-β-stimulated expression of collagen type I and plasminogen activator inhibitor-1, improves renal vascular endothelial cell function, and improves renal fibrosis.^[28]^ Hemodynamic abnormalities are the early manifestations of diabetic microcirculation disorders. By regulating vascular tone, improving blood flow and reducing blood flow resistance, GFR and urinary albumin excretion rate will return to normal.

### 3.2. Microangiopathy

Microangiopathy is the basis of various complications of diabetes. The thickness of glomerular basement membrane (GBM), the expansion of glomerular mesangial cells (GMC) and the accumulation of GBM are the typical pathological features of DN. GBM and renal utricle epithelial cells, also known as podocytes, are critical components of the filtration barrier.^[29]^ The increased excretion of large amounts of nonselective proteinuria in DN patients means that hemodynamic changes in the nephron lead to hyperfiltration and activation of the RAAS, and the mechanical barrier structure of the filtration membrane is damaged. At this time, the glomerular lesion has developed to be irreversible.^[30]^ It can improve proximal renal tubular changes and glomerular filtration rate by regulating endothelial cell function, restore glomerular endothelial barrier function, and reduce the permeability of filtration membrane.^[31]^ Glomerular podocyte injury is considered to be one of the main mechanisms leading to proteinuria. Studies have shown that thioredoxin interacting protein – mediated oxidative stress activates NOD-like receptor protein 3 (NLRP3) inflammasome and promotes the maturation and secretion of Caspase-1. Induced podocyte pyroptosis.^[32]^ Previous studies have confirmed that it has a definite protective effect on the kidneys of DN model rats, covering multiple dimensions such as oxidative stress, regulation of cell death, and pathological morphology repair. By regulating the Txnms-NLRP3-GSDMD signaling axis, it significantly inhibits renal cell pyroptosis by suppressing the activation of NLRP3 inflammasome and GSDMD-mediated pore formation, reduces the pro-inflammatory programmed cell death of renal parenchymal cells, down-regulates the expression of apoptosis-related proteins Caspase-3 and Bax, upregulates the level of anti-apoptotic protein Bcl-2, protects the number and functional integrity of podocytes, thereby maintaining the stability of the glomerular filtration barrier and effectively reducing the generation of proteinuria.^[33]^

GMC can secrete ECM proteins, including collagen IV, laminin and fibronectin, which play important roles in the physiology and pathology of the kidney. The abnormal expression of TGF-β and nuclear factor-кB proteins in GMCS promotes renal cell hypertrophy and stimulates ECM accumulation, and mediates glomerular and interstitial fibrosis in DN.^[34]^ Continuous hyperglycemia can induce the proliferation of GMC and the increase of TGF-β expression, which may lead to diabetic kidney damage by promoting the synthesis of ECM-related proteins or inhibiting the degradation of proteins.^[35]^ It can directly inhibit the proliferation of the main pathogenic target cells of DN-GMC cells, significantly upregulate the expression level of type I TGF-β receptor in renal fibroblasts, and the upregulation of this expression can enhance the signal response ability of renal fibroblasts to TGF-β, thereby activating and strengthening the TGF-β signaling transduction activity.^[36]^ At the same time, it also inhibits the expression of ECM proteins to prevent and delay the development of renal fibrosis.^[37]^

### 3.3. Hemorheological changes

Hemorheology is the main manifestation of diabetic microcirculation disorder, and it is also one of the important factors causing the continuous development of diabetic kidney disease. It is clinically characterized by increased blood viscosity, decreased flow rate, enhanced aggregation and weakened deformability of red blood cells. Renal microangiopathy is characterized by decreased blood flow of peritubular capillaries in renal tubules, resulting in chronic tissue ischemia and hypoxia, resulting in renal function damage.^[38,39]^ Renal microvascular disease observed in the early stage of diabetic nephropathy (CKD stage 1–2) may progress to advanced diabetic nephropathy (CKD stage 3–5) with impaired endothelial factors, altered vascular homeostasis, and a further reduction in perirubular capillary flow caused by an imbalance of PGI_2_-TXA_2_.^[40]^

It promotes the proliferation of endothelial cells and the expression of cell adhesion molecules, and then regulates the formation of red blood cells through hematopoietic progenitor cells. Glutamate-glutamine metabolism is an important way to promote the formation of red blood cells and related cells.^[41]^ In addition, renal erythropoietin (EPO), hypoxia-inducible factor-1 (HIF-1), a key genetic component in the fibrotic process of diabetic nephropathy, was found to induce changes in cell type-specific gene expression, increase renal EPO production, and regulate the bone marrow microenvironment to promote erythroid progenitor cell maturation and proliferation. Due to renal hypoxia caused by renal microcirculation disorders in DN patients, increased glucose reabsorption creates a high glucose environment in the renal tubulointerstitium, which may impair HIF-1 and renal EPO-producing cells and reduce EPO secretion and erythropoiesis.^[42,43]^ Oxidative stress caused by tissue ischemia-reperfusion can cause abnormal aggregation and immunosuppressive ability of erythroid progenitor cells by inhibiting the transcription inhibitory factors Pu.1 and Gata-1, inhibit the differentiation of epcs into mature red blood cells, and destroy the homeostasis of hematopoietic function.^[44]^ The transcription factors Pu.1 and Gata-1 can alleviate abnormal erythroid progenitor cell accumulation, enhance antitumor immune response, and effectively alleviate the further development of renal microcirculation disorders.^[45]^

## 4. Signaling pathways associated with renal microcirculation

DN is a fatal complication of diabetes and the leading cause of ESRD. The main pathological features of ESRD are glomerular basement membrane thickening, mesangial cell proliferation and extra-mesangial matrix proliferation. Podocytes also play an extremely important role in the development of DN.^[46]^ More and more studies have shown that DN is caused by a variety of microcirculation disorders, including oxidative stress, a variety of inflammatory factors, glucose and lipid metabolism disorders, and immune dysfunction. The abnormalities and interactions of a variety of signaling pathways jointly promote the occurrence and development of DN.^[47-49]^

### 4.1. MAPK signaling pathway in DN

**Figure 2.**
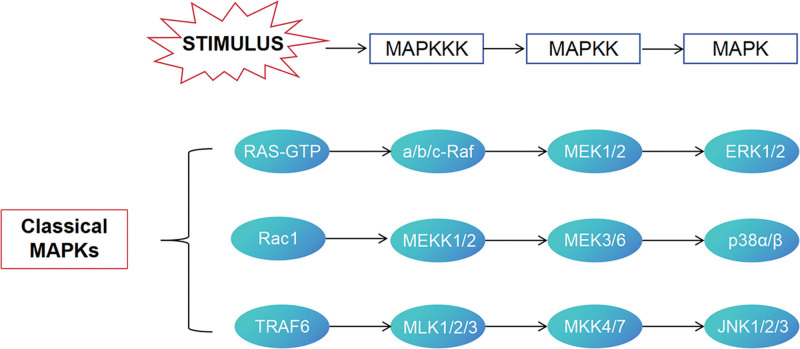
The MAPK signal transduction pathway in DN. DN = diabetic nephropathy, MAPK = Mitogen-activated protein kinase.

The signal transduction of mitogen-activated protein kinases (MAPKs) is composed of a group of serine/threonine protein kinases, which mediate cellular information transmission through MAPKKK-MAPKK-MAPK amplification cascade activation and participate in different pathways to jointly accelerate the progression of DN.^[50]^ The specific mechanism of action is shown in Figure 2. Extracellular regulated protein kinases (ERK), a member of the MAPKs family, are activated in mesangial cells exposed to high glucose and in the glomeruli of diabetic rats dependent on the activation of PKC. In mesangial cells exposed to PKC inhibitors, the activation of ERK is abolished.^[51]^ Under high glucose condition, PKC activation not only enhances NADPH oxidase activity, but also promotes ROS production in endothelial cells and mesangial cells, which leads to renal microangiopathy and cell damage in renal tissue.^[52,53]^ The increased expression of PG and NO in renal tissue can induce renal hemodynamic imbalance and aggravate the damage of renal function and structure on the basis of the original injury.^[54]^ Some studies have shown that PKC activation is inhibited by reducing the phosphorylation of JNK and p38, reducing the levels of ERK, p38MAPK, JNK and other family kinases, and preventing the non-phosphorylation and phosphorylation of PKC isoforms.^[55]^ p38 MAPK and JNK MAPK are key intracellular signal transduction pathways involved in the production of inflammatory mediators, which have been confirmed to be activated in glomeruli and tubules. The activation of p38 MAPK and JNK MAPK in podocytes and glomerular endothelial cells further suggests that MAPK pathway plays an important role in the process of renal fibrosis.^[56]^ With the deepening of research, it has been found that persistently elevated blood glucose can be used as a key stimulator to promote the expression of AGEs receptors in glomerular epithelial cells, mesangial cells, endothelial cells and podocytes. After AGEs interact with their receptors, they trigger an increase in ROS levels in endothelial cells, thereby initiating the oxidative stress process. On this basis, oxidative stress can mediate the abnormal activation of the downstream JNK and p38-MAPK signaling pathways, leading to a significant increase in the release of adhesion molecules, vascular endothelial factors and inflammatory factors, and ultimately aggravate the pathological damage of the kidney.^[57,58]^ Studies have found that p38 MAPK is activated under high glucose stimulation, and its activation can mediate the phosphorylation of nuclear factor-κB inhibitor α, and then promote the activation of NF-κB. Activated NF-κB can induce the proliferation of GMCS and inhibit the abnormal accumulation of ECM in GMCS by interfering with the normal function of matrix related proteins, so as to prevent the thickening of GBM, which will lead to the prolonged progression of DN.^[59]^

### 4.2. TGF-β signaling pathway in DN

**Figure 3.**
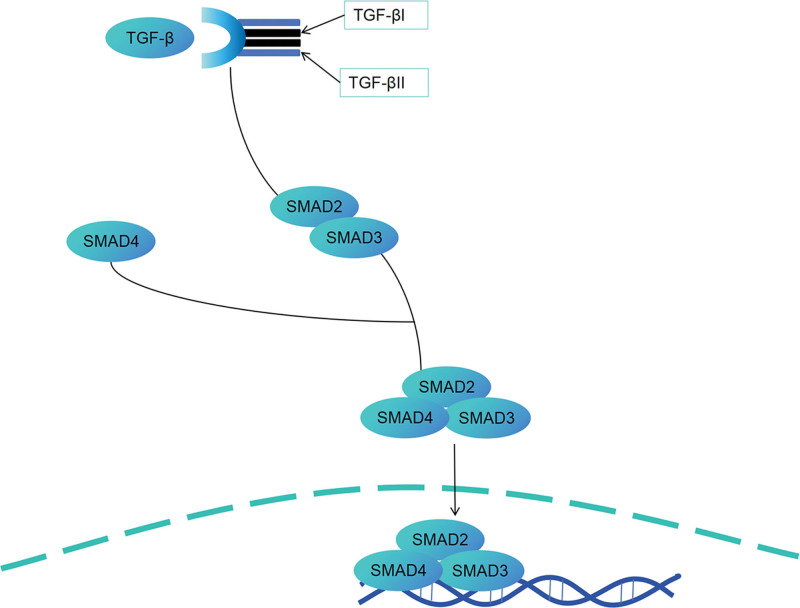
The TGF-β signal transduction pathway in DN. DN = diabetic nephropathy, TGF-β = transforming growth factor-β.

The TGF-β family, as an important cellular signal regulatory factor, plays a central role in the proliferation of cells, the formation of cell matrix, the regulation of immune function, and the regulation of inflammatory responses in the body. Abnormal functions of this family are closely related to the pathological progression of various kidney diseases, especially DN. In the pathogenesis of DN, TGF-β1 (the key subtype of the TGF-β family) is the core molecule that drives glomerular sclerosis and renal interstitial fibrosis. Its pathogenic effect is mainly achieved through the following pathways: On one hand, TGF-β1 can activate downstream signaling pathways (such as the Smad signaling pathway), causing pathological hypertrophy of glomerular mesangial cells, glomerular endothelial cells, and renal tubular epithelial cells; on the other hand, TGF-β1 can significantly upregulate the synthesis and secretion of membrane matrix proteins such as collagen (types I, III, and IV), fibronectin, and laminin in glomerular and renal tubular cells, while inhibiting the activity of matrix-degrading enzymes such as matrix metalloproteinases, leading to excessive deposition of ECM in the glomerular mesangial area, glomerular basement membrane, and renal interstitium, ultimately causing glomerular sclerosis and renal interstitial fibrosis, and resulting in progressive decline of renal function. Further experimental studies have further confirmed the pathogenic role of TGF-β1 in DN: In diabetic model rats, the expression level of TGF-β mRNA in the renal glomerular tissue significantly increased, and the expression level was positively correlated with the degree of glomerular sclerosis and interstitial fibrosis in the kidneys; more direct evidence came from intervention experiments – injecting exogenous TGF-β into the subcutaneous area of normal rats, it could be observed that obvious collagen deposition and fibrotic changes occurred in the renal tissue, and the pathological manifestations were highly similar to the kidney damage of DN model rats. The above studies fully demonstrate that the abnormal high expression of TGF-β1 is the key driving factor in the pathological process of renal fibrosis in DN.^[60]^ Hyperglycemia and oxidative stress stimulate the expression of TGF-β, which is the most important cytokine regulating the formation/degradation of ECM, mesangial expansion and glomerular basement membrane thickening. Down-regulating the expression levels of TGF-β and type Ⅳ collagen in renal tissue can effectively improve the pathological changes of diabetic kidney in the early stage of the disease.^[61]^ Evidence has shown that the generation of interstitial myofibroblasts during TGF-β-mediated epithelial-mesenchymal transition (EMT) is also a key factor in the process of fibrosis. When TGF-β binds to its receptor, it activates downstream signal transduction molecules (SMAD2 and SMAD3), which ultimately mediate the occurrence and development of fibrosis.^[62,63]^ The specific mechanism of action is shown in Figure 3. TGF-β1 also promotes podocyte apoptosis, destroys the mechanical barrier structure of the filtration membrane and leads to albuminuria. At the same time, Tgf-β1 and activated NF-κB work together to reduce the activity of ECM-related protein degrading enzymes, increase the production of inhibitors of protease activity, and aggravate protein matrix aggregation. The abnormal structure of renal filtration membrane barrier can be effectively reversed or alleviated by reducing the proliferative activity and down-regulating the protein expression of TGF-β1 and NF-κB in GMC cells, which is the key pathogenic target of DN. This regulation is of great significance in the protection of DN kidney.^[64,65]^ Bone morphogenetic protein-7 (BMP-7) belongs to the TGF-β superfamily, but has a negative regulatory effect on renal fibrosis, inhibiting the apoptosis of renal epithelial cells and regulating epithelial-mesenchymal transition (EMT). The alcohol extract of Angelica Buxue decoction significantly increased the expression of BMP-7 protein and mRNA in renal tissue. It can affect the TGF-β1/Smads transduction pathway and alleviate the imbalance of ECM accumulation and degradation.^[66]^ It is worth noting that TGF-β1 is also involved in the activation of MAPK downstream signaling pathways, including ERK, JNK and p38 MAPK. Studies have confirmed that these MAPK downstream pathways activated by TGF-β1 regulation act synergistically in multiple links of DN pathogenesis to jointly promote disease progression.^[67]^

### 4.3. JAK/STAT signaling pathway in DN

**Figure 4.**
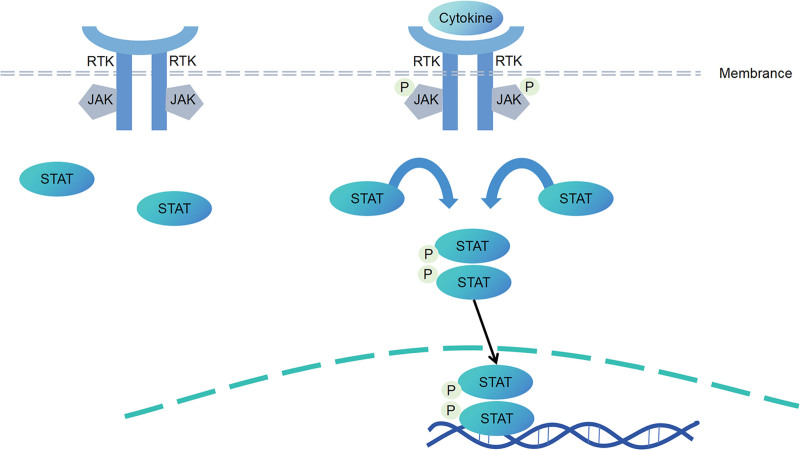
The JAK/STAT signal transduction pathway in DN. DN = diabetic nephropathy, JAK/STAT = janus kinase-signal transducer and activator of transcription.

The activation of janus kinase-signal transducer and activator of transcription (JAK/STAT) pathway plays an important role in the pathogenesis of DN. Under high glucose conditions, JAK-2 and STAT-3 are activated, while TGF-β and fibronectin are synthesized in glomerular mesangial cells. However, when JAK and STAT tyrosine phosphorylation is inhibited, TGF-β and fibrin synthesis are also inhibited. It is not difficult to observe that the JAK-2/STAT-3 pathway can be activated by hyperglycemia, and the activated pathway plays a key role in promoting the proliferation of glomerular GMC and the synthesis of ECM proteins.^[68]^ Studies have confirmed that high glucose conditions can drive mesangial cells to produce a large amount of ROS and increase the expression level of Nrf2. The above changes will trigger the downstream molecular cascade, which is manifested as the phosphorylation of JAK2 and STAT3, leading to the activation of JAK2/STAT3 pathway.^[69]^ The role of JAK/STAT pathway is not limited to renal intrinsic cells, but also regulates the proliferation of extrarenal cells. Specifically, with the induction of AGEs, this pathway promotes mitosis of NRK-49F cells and leads to abnormal increase in cell proliferation, which ultimately becomes an important driver for the development of renal fibrosis.^[70]^ In addition, JAK/STAT pathway can regulate gene expression, cell activation, proliferation and differentiation, as well as EMT and fibrosis in DN.^[71]^ In the local microenvironment of the kidneys in DN patients, the imbalance of macrophage polarization (with an increase in the proportion of M1 type pro-inflammatory macrophages and a weakened function of M2 type anti-inflammatory macrophages) is an important factor that exacerbates kidney inflammation and promotes glomerular damage. Moreover, M2 type macrophages, as a subtype of macrophages with anti-inflammatory and tissue repair functions, play a crucial regulatory role in improving the pathological process of DN. The M2 type macrophages can inhibit the abnormal activation of JAK2 and STAT3 in GMCs through paracrine cytokines (such as anti-inflammatory factors like IL-10 and TGF-β1) or direct cell-to-cell interactions. The related intervention studies further confirmed the protective effect of M2-type macrophages. By repeatedly injecting exogenous M2-type macrophages into DN model animals, it not only effectively corrected the local imbalance of M1/M2 macrophages in the renal glomeruli (reducing the infiltration ratio of M1-type macrophages and enhancing the anti-inflammatory function of M2-type macrophages), but also continuously inhibited the activation level of the JAK2/STAT3 pathway in GMCs. The final result was: the expression of pro-inflammatory factors (TNF-α, IL-6) in renal tissue was significantly reduced, and the inflammatory infiltration in the glomeruli was significantly alleviated; at the same time, the degree of glomerular mesangial proliferation was relieved, ECM deposition was reduced, effectively delaying the progression process of DN from early mesangial damage to glomerular sclerosis, providing important experimental evidence for the immunological regulation therapy of DN.^[68]^ The mRNA expression of JAK members is upregulated in the early stages of DN and downregulated in progressive DN. Activation of the JAK/STAT pathway is an important mechanism of renal injury caused by hyperglycemia, and regulation of this pathway can effectively prevent renal and vascular complications of diabetes. Experiments have found that inducing diabetes leads to JAK/STAT activation and increased expression of SOCS1 and SOCS3. These results suggest that there is a direct correlation between JAK/STAT/SOCS axis and renal cell injury induced by high glucose. JAK/STAT/SOCS axis plays a protective role in the process of renal injury induced by high glucose by reducing renal cell apoptosis and providing renal cell injury protection^[72]^ (Fig. 4).

### 4.4. Other signaling pathways in DN

The oxidative stress response is one of the earliest critical events that occur during the pathological progression of DN. Its abnormal activation persists throughout the entire course of DN, especially during the early stage of the disease, where it participates in the initiation and amplification of kidney damage, laying the pathological foundation for subsequent glomerular, tubular damage and renal function decline. In the early stage of DN, persistent hyperglycemia is the core trigger for inducing an imbalance in oxidative stress: the high sugar environment can disrupt the homeostasis of the body’s oxidative and antioxidant systems through multiple pathways (such as abnormal mitochondrial respiratory chain function, excessive activation of the polyol pathway, increased production of advanced glycation end products, etc) – a large amount of ROS (such as superoxide anion, hydrogen peroxide, etc) is abnormally generated; in addition, the activities of endogenous antioxidant enzymes such as SOD and GSH-Px are inhibited, leading to a large accumulation of ROS in kidney tissues (especially vascular endothelial cells). The excessive accumulation of ROS directly causes damage to renal vascular endothelial cells, and the mechanisms include: first, ROS can attack the lipid components of the endothelial cell membrane, triggering lipid peroxidation reactions, destroying the integrity and fluidity of the cell membrane structure, leading to dysfunction of the endothelial cells; second, ROS can oxidatively damage DNA and protein molecules within the endothelial cells, affecting normal metabolism and signal transduction, inhibiting eNOS activity, reducing NO production, and weakening vascular dilation ability; third, damaged endothelial cells will further release pro-inflammatory factors and adhesion molecules (such as IL-6, VCAM-1), exacerbating local inflammation in the kidneys, while damaging the vascular barrier function, laying the groundwork for the occurrence of subsequent glomerular hyperfiltration, microalbuminuria and glomerular sclerosis.^[73]^ Oxidative stress and its associated inflammation lead to pathological damage of podocytes, endothelial cells, mesangial cells and renal tubular epithelial cells.^[74]^ By inhibiting the NOX-4/ROS/p38 pathway, oxidative cell damage was alleviated and Bcl-2 family related podocyte apoptosis was inhibited.^[75]^ In addition, regulation of the ROS-mediated PI3K/AKT pathway reduced 24-hour urinary protein, serum creatinine and blood urea nitrogen levels in DN mice, demonstrating that the formula could reduce proteinuria and alleviate podocyte injury.^[76]^ Excessive ROS can also activate PKC, resulting in the increase of advanced glycation end products, leading to glomerular fibrosis. The overexpression of ROS can also induce the initiation of autophagy, and the defect of autophagy can cause the imbalance of energy homeostasis and the disorder of glucose and lipid metabolism. It is worth noting that autophagy can regulate the function of pancreatic β-cells and the physiological state of insulin-targeted tissues. Through experiments, inhibiting autophagy leads to aggravated damage to podocytes, thereby promoting the development of DN and accelerating the progression of the disease.^[77]^ Autophagy plays an important role in the maintenance of podocyte homeostasis. Ursolic acid inhibits the expression of miR-21 and increases the expression of PTEN, which in turn inhibits Akt and mTOR and restores the normal level of autophagy, significantly improves renal function and reduces mesangial proliferation in DN mice. It indicates that ursolic acid regulates the PI3K/Akt/mTOR signaling pathway under pathological conditions, thereby affecting the autophagy of renal podocytes and protecting the kidney to delay the development of DN.^[78,79]^ Oxidative stress can also lead to mitochondrial dysfunction. Excessive ROS expression leads to the activation of NLRP3 inflammasome and the upregulation of pro-inflammatory factors, which aggravates glomerular dysfunction in DN. NLRP3 inflammasome aggravates lipid accumulation in diabetic nephropathy and leads to podocyte injury.^[80]^ Triptolide (TP) inhibits pyroptosis through Nrf2/ROS/NLRP3 axis, alleviates oxidative stress and thus reduces the release of inflammatory factors and proteinuria, thus exerting DN effect.^[81,82]^

## 5. Conclusion and discussion

DN is a specific microvascular lesion induced by persistent hyperglycemia. One of its core pathophysiological mechanisms is renal microcirculation disorder, which not only runs through the entire course of DN but is also a key driving factor for abnormal glomerular filtration function and progressive decline in renal function. Currently, treating DN by improving renal microcirculation abnormalities shows potential value. Microcirculation disorders include hemodynamic abnormalities, microvascular lesions, and changes in hemorheology. The mechanisms include: regulating the function of renal vascular endothelial cells to balance the contractile tension of the afferent and efferent arterioles of the glomerulus, thereby reducing intraglomerular hypertension and increasing renal blood flow; at the same time, it can alleviate the early state of high filtration in the glomerulus, reduce the resulting microalbuminuria, and delay the progression of renal function impairment. From the molecular mechanism perspective, by activating the PI3K/Akt signaling pathway, promoting phosphorylation and activation of eNOS, and upregulating eNOS activity to accelerate NO production; It can also regulate PG intermediate metabolites, restore the balance of PGI_2_ and TXA_2_, improve vascular endothelial cell permeability, and repair vascular damage caused by disrupted renal cell function. In the early stage of DN, RAAS plays an indispensable role in maintaining renal hemodynamic stability (such as arterial pressure regulation, tissue perfusion guarantee, and extracellular fluid volume control), and AngⅡ, as the core effector molecule of RAAS, upregulates the expression level of TGF-β mRNA in glomerular mesangial cells, promoting the synthesis of ECM components such as fibronectin, collagen, and laminin, resulting in excessive deposition of ECM in the glomerulus and renal interstitium; on the other hand, AngⅡ can directly damage glomerular podocytes and disrupt the integrity of the glomerular filtration barrier. However, there are obvious gaps in current research, and systematic studies on regulating RAAS to improve hemodynamic abnormalities for the treatment of DN are still relatively scarce. There is also a lack of sufficient experimental evidence to support the specific regulatory effects and molecular mechanisms of renal microcirculation disorders. Most related studies focus on the role of AngⅡ in inducing apoptosis of rat myocardial cells and arterial endothelial cells, while there are few reports on the specific mechanisms of AngⅡ and other components of RAAS in diabetic renal microvascular lesions (such as glomerular capillary loop occlusion, abnormal endothelial cell function), and this field still has considerable research potential for exploration. Renal microvascular lesions are the hallmark pathological feature of DN. Under the continuous stimulation of high glucose, multiple cellular signaling pathways are abnormally activated, and these pathways act alone or in synergy to jointly promote the occurrence and progression of DN. TGF-β plays a “core regulator” role in renal injury in DN, participating in glomerular sclerosis and renal interstitial fibrosis processes by regulating cell proliferation and ECM formation: on one hand, TGF-β can inhibit excessive proliferation of GMC, reduce the accumulation of ECM components such as fibronectin and laminin, theoretically having the potential to reverse early glomerular sclerosis; on the other hand, TGF-β1 can activate downstream molecules of MAPK (such as ERK, p38 MAPK), further exacerbating renal damage by regulating cell signal transduction. In a high-glucose environment, the ERK and p38 MAPK pathways are activated, not only promoting GMC proliferation and excessive deposition of ECM, but also reducing ERK expression by inhibiting PKC activation, and enhancing the translation efficiency of HIF-1α, improving the ischemic state of renal tissue. p38 MAPK can also form a “cross-regulation” with the NF-κB inflammatory pathway: activated p38 MAPK can further activate NF-κB, promoting the expression of matrix metalloproteinases in mesangial cells, affecting the balance of ECM metabolism; On the other hand, it stimulates the release of pro-inflammatory factors such as TNF-α and IL-6, intensifying the local inflammatory response in the kidneys and forming a vicious cycle of “inflammation-ECM deposition,” accelerating the progression of DN. The activation of PKC is an important “bridge” between oxidative stress and kidney damage in DN: The activation of PKC can exacerbate the oxidative stress response and promote excessive ROS generation; while the accumulation of ROS not only promotes the formation of AGEs (AGEs are the key pathogenic factors causing kidney fibrosis), but also activates the JAK/STAT signaling pathway, upregulating the synthesis of TGF-β and fibronectin in GMC, further aggravating ECM deposition. In addition to these mechanisms, ROS can also affect the progression of DN through multiple pathways: First, ROS is closely related to autophagy reactions, which can affect the metabolic disorder of sugars and lipids in DN by regulating autophagy activity; Second, ROS has a targeted damaging effect on pancreatic β-cells, reducing insulin secretion capacity and indirectly exacerbating the hyperglycemic state, forming a vicious cycle of “high sugar-ROS-pancreatic damage,” further deteriorating renal microcirculation and renal function.

Based on the current research status, there are still 2 core directions that urgently need in-depth exploration. First the precise regulatory mechanism of renal microcirculation-related signaling pathways. Currently, the “synergistic regulatory network” of TGF-β, MAPK, JAK/STAT and other pathways in renal microcirculation disorders has not been fully clarified, and it is necessary to further clarify the interaction nodes between each pathway to provide more precise molecular targets for targeted improvement of microcirculation. Second, the material basis for improving renal microcirculation, whether it is drugs or other intervention methods, the “key active ingredients” and “target molecules” for improving renal microcirculation in DN are still unclear. Through component separation, molecular docking, in vitro and in vivo verification and other studies, the material basis and mechanism of action need to be clarified to provide theoretical support for the development of DN microcirculation intervention drugs.

## Acknowledgments

The authors acknowledge the support of Siyao Deng and Changyu Gao (literature review).

## Author contributions

**Conceptualization:** Weikang Tang, Siyao Deng.

**Funding acquisition:** Huixia Liu, Xuan Li, Siyao Deng.

**Supervision:** Huixia Liu, Xuan Li, Changyu Gao.

**Writing – original draft:** Weikang Tang.

**Writing – review & editing:** Weikang Tang, Siyao Deng, Changyu Gao.
